# A false aneurysm with an aorto-superior vena cava fistula after replacement of the ascending aorta: report of a case

**DOI:** 10.1007/s00595-013-0723-1

**Published:** 2013-10-02

**Authors:** Ryuma Iwaki, Keitaro Nakagiri, Naoto Morimoto, Hirohisa Murakami, Tasuku Honda, Masato Yoshida, Nobuhiko Mukohara

**Affiliations:** Division of Cardiovascular Surgery, Department of Surgery, Hyogo Brain and Heart Center at Himeji, 520 Saisho-kou, Himeji, 670-0981 Hyogo Japan

**Keywords:** Aortic operation, CHD, Valve, CABG, Venous grafts

## Abstract

A 58-year-old female presenting with congestive heart failure due to a fistula between an aortic false aneurysm and the superior vena cava (SVC) is described. She had a history of Takayasu’s arteritis (TA) and she had undergone aortic valve and ascending aorta replacement and coronary artery bypass grafting 6 years before. The false aneurysm had occurred 1 year after the surgery, and she had been conservatively managed. The operation revealed that the cause of the false aneurysm was the detachment of the two proximal saphenous vein anastomoses to the ascending aortic graft. After the surgery, the patient made an uneventful recovery. A false aneurysm of the ascending aorta is one of the most serious complications after replacement of the ascending aorta for patients with TA (Miyata et al. in J Vasc Surg 27:438–445, 1998). We herein present the exceptional case of a fistula between an aortic false aneurysm and the SVC that occurred after ascending aorta graft replacement.

## Case report

A 58-year-old female who was diagnosed to have TA when she was 30 years old had presented with dyspnea on exertion 6 years ago. She was diagnosed with aortic regurgitation with dilatation of the ascending aorta and stenosis of the left main coronary artery. At that time, aortic valve replacement (SJM standard 27 mm) and ascending aorta replacement (Intervascular^®^ 28 × 10 mm) and coronary artery bypass grafting (bypass to the left ascending and circumflex artery with vein grafts from the ascending prosthetic graft) were performed. After the surgery, she was administered 15 mg prednisolone/day. One year after the surgery, she had developed an ascending aorta false aneurysm and had been advised to have surgery. However, the patient had refused surgery and had been conservatively managed.

**Fig. 1:**
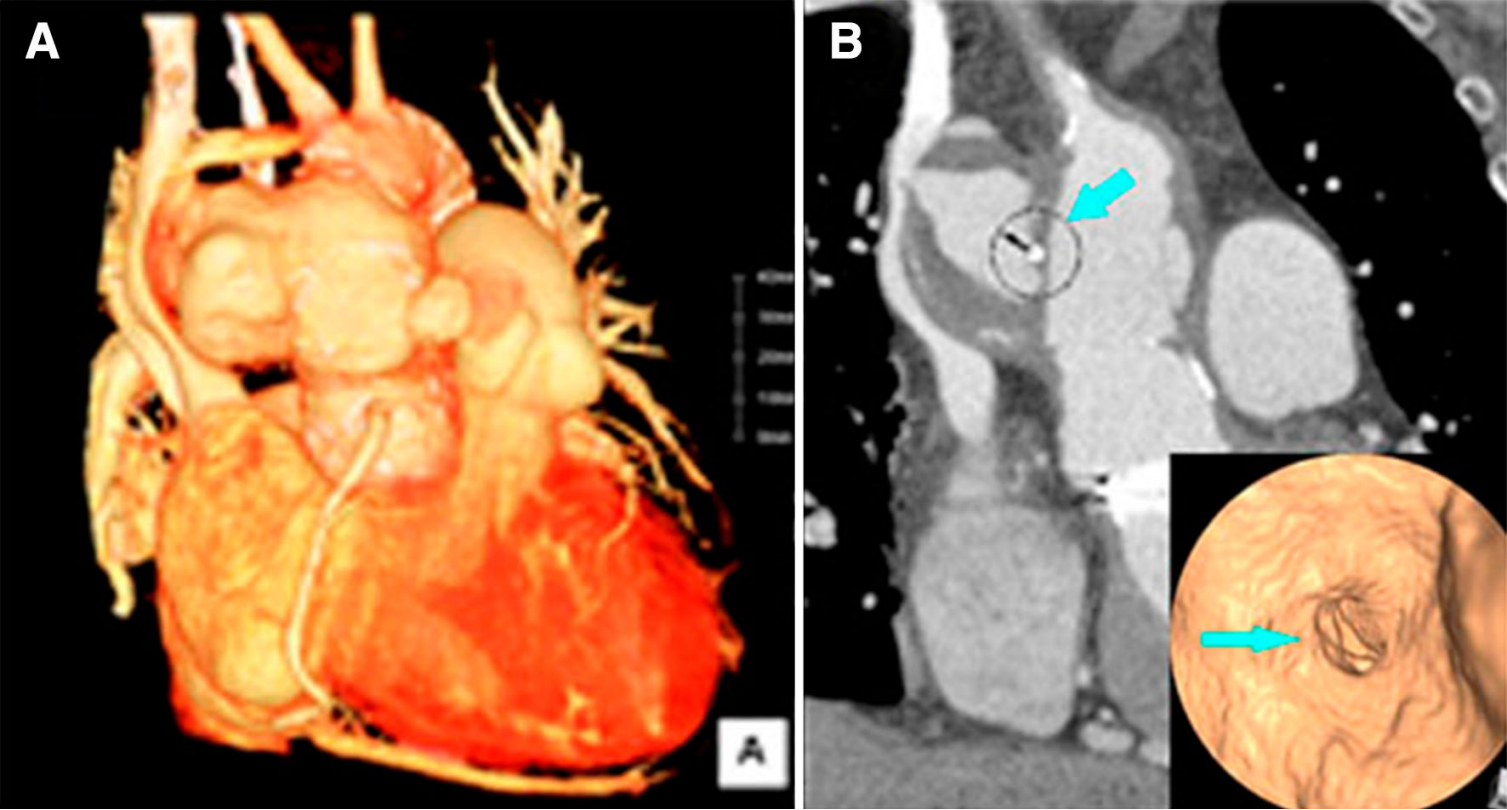
**a** A 73 × 44 mm large false aneurysm with an irregular shape was found, and the false aneurysm compressed the superior vena cava (SVC). **b** Coronal CT and a virtual CT endoscopic view showed the prosthetic graft punch hole (*arrows*)

At this presentation, she showed orthopnea and peripheral edema. Auscultation revealed a continuous diastolic–systolic murmur and diminished basilar breath sounds. Chest radiography showed bilateral pleural effusions and interstitial fluid accumulation, and the cardiothoracic ratio was 0.62. Laboratory examinations showed a high white blood cell count (11.6 × 10^3^/μL) and an elevated C-reactive protein level (1.4 mg/dL). The brain natriuretic peptide level was also high (933). Echocardiography demonstrated the shunt flow between the ascending graft and the false aneurysm. A contrast-enhanced computed tomography (CT) scan showed a 73 × 44 mm large false aneurysm with an irregular shape, and the false aneurysm compressed the superior vena cava (SVC) (Fig. 1). Aortography confirmed the presence of an aorto-SVC shunt (pulmonary/systemic perfusion ratio of 2.23). Coronary angiography showed no significant stenosis and occlusion even at the previous stenotic lesions.

**Fig. 2:**
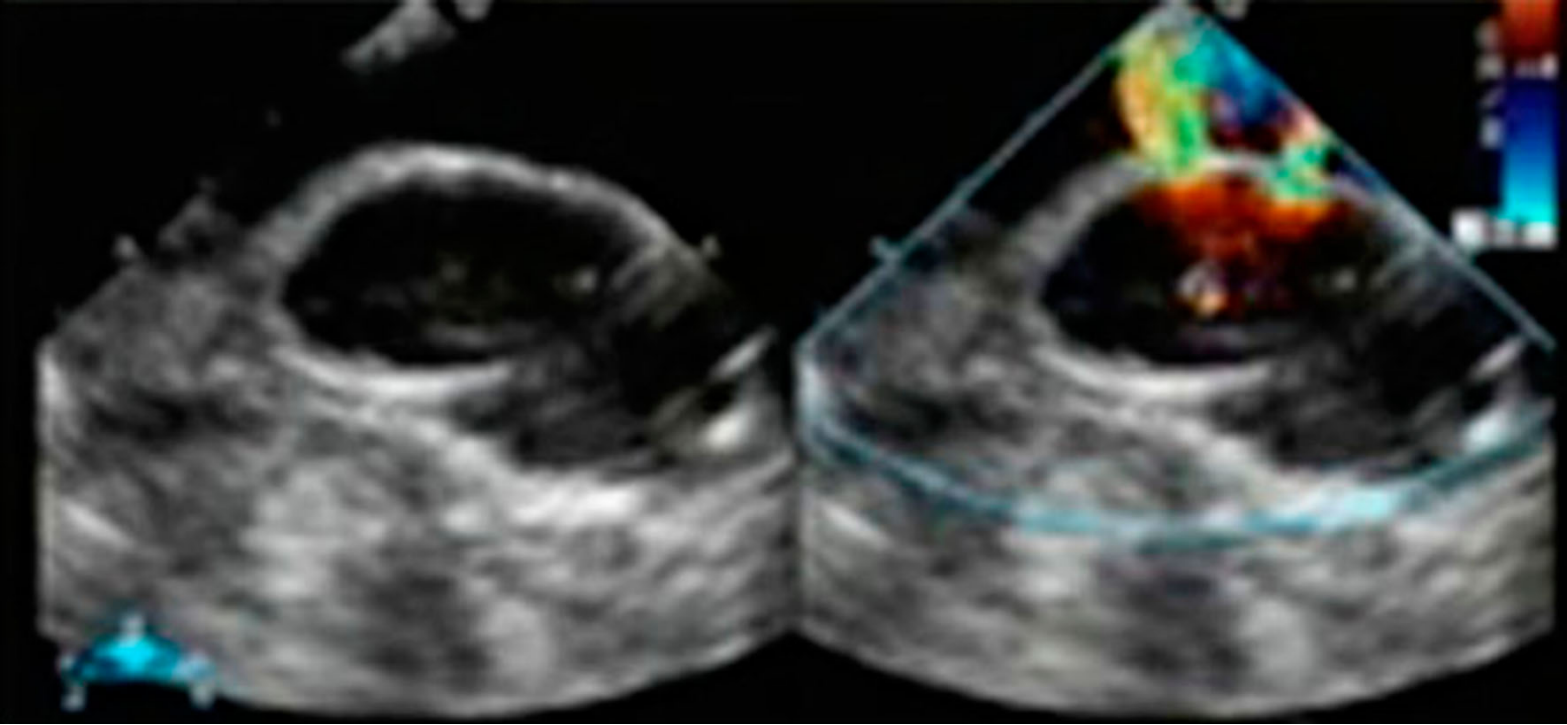
Epi-aortic echocardiography showed shunt flow from the two punch holes of the proximal anastomoses at the CABG

During surgery, cardiopulmonary bypass with cannulation of the right femoral artery and vein was instituted before sternotomy. Re-sternotomy was performed uneventfully. Epi-aortic echocardiography showed shunt flow between the ascending aorta and the false aneurysm through two punch holes of the proximal anastomoses at the CABG (Fig. 2). Systemic cooling was performed to a rectal temperature of 28 °C. Under circulatory arrest, the false aneurysm was incised, and we found two prosthetic graft punch holes, while there were no vein grafts in the false aneurysm. We closed the prosthetic graft punch holes directly. The false aneurysm had perforated to the SVC and there was a 25 × 10 mm defect between the SVC and false aneurysm. We closed the defect of the SVC with a bovine pericardium patch. We did not perform re-CABG, because there were no stenotic lesions in her native coronary arteries. The patient made a good postoperative recovery, and the peripheral edema diminished rapidly. Contrast-enhanced CT showed good patency of the SVC. There was no evidence of infection from the false aneurysmal tissue samples.

## Discussion

An anastomotic false aneurysm of the ascending aorta is one of the most serious complications after replacement of the ascending aorta for patients with TA. In the largest series from Japan, the incidence of anastomotic false aneurysm was 8.5 % [1]. Ogino et al. [2] reported that in recent cases, 1.8 % of the patients at 10 years and 3.5 % at 20 years had developed anastomotic false aneurysms.

The reported causes of false aneurysms are wall weakness, compliance mismatch between the host artery and implanted graft, systemic inflammatory reactions and steroid administration. In our case, the proximal and distal anastomosis between the ascending aorta and the prosthetic graft were normal. The origin of the false aneurysm was punch holes caused by the detachment of the proximal anastomosis between the SVG and prosthetic graft. We considered that the continuous use of a steroid for TA might have induced tissue fragility, and that tissue dehiscence had occurred. However, Miyata et al. reported that systemic inflammatory reactions (*P* = 0.87) and steroid administration (*P* = 0.55) had little influence on the formation of anastomotic false aneurysms, and they suggested that the type of lesion (aneurysmal or occlusive) was the most significant factor. False aneurysms occurred 4.85 times more often in aneurysmal lesions than in occlusive lesions [1]. They also suggested that an anastomotic aneurysm can occur at any time after surgery [1, 3].

False aneurysms of the ascending aorta occur in <0.5 % of all cardiac surgical cases, and are rare except for cases with inflammatory aortic disease, like aortitis [4]. The predominant predisposing factors for false aneurysms of the aorta are aortic graft infection or dissection of the aorta, trauma, predisposing degenerative aortic disease, in addition to several other reasons [5, 6]. In our case, there was no evidence of risk factors for the development of a false aneurysm, except for TA and steroid administration.

Most cases of false aneurysm of the ascending aorta are detected due to symptoms of congestive heart failure, chest pain and sepsis. Asymptomatic cases are comparatively rare [4, 5]. In our case, an asymptomatic false aneurysm of the ascending aorta was incidentally detected after 1 year of the previous surgery, and had been asymptomatic for 6 years. However, the false aneurysm had gradually enlarged and symptoms of congestive heart failure had developed after the formation of the aorto-SVC fistula. There have been several reports of aorto-right heart fistulas with an enlarged false aneurysm of the ascending aorta. Most reported cases involved the aorto-pulmonary artery, aorto-right atrium, and rarely, the aorto-right ventricular fistula. To our knowledge, this is the second case of an aorto-SVC fistula.

In conclusion, a false aneurysm of the ascending aorta after cardiovascular surgery for patients with TA is not rare, and it can occur at any time after surgery. Therefore, patients should be carefully monitored after the initial surgery. Surgery is the only curative treatment for a false aneurysm, and once a false aneurysm is detected, surgical intervention should be undertaken as soon as possible.
